# Chemical composition and antibacterial activity of oils from *Chrysicthys nigrodigitatus* and *Hepsetus odoe,* two freshwater fishes from Yabassi, Cameroon

**DOI:** 10.1186/s12944-018-0690-z

**Published:** 2018-03-12

**Authors:** Mouokeu Raymond Simplice, Womeni Hilaire Macaire, Njike Ngamga Fabrice Hervé, Tonfack Djikeng Fabrice, Djopnang DJimbie Justin, Tchoumbougnang François, Kuiate Jules-Roger

**Affiliations:** 1 Laboratory of Fisheries Resources, Institute of Fisheries and Aquatic Sciences, University of Douala, Po Box 7236, Douala, Cameroon; 2 Laboratory of Biochemistry of Medicinal Plants, Food Sciences and Nutrition, University of Dschang, Po Box 67, Dschang, Cameroon; 3 Laboratory of Microbiology and Antimicrobial Substances, University of Dschang, Faculty of Science, Po box 67, Dschang, Cameroon

**Keywords:** *Chrysichthys nigrodigitatus*, *Hepsetus odoe*, Fish oil composition, Antibacterial activity

## Abstract

**Backgrounds:**

Oils of fish origin are a very rich source of Omega − 3 and Omega − 6 fatty acids. They have been suggested to provide numerous health benefits for humans involving antimicrobial properties. *Chrysichthys nigrodigitatus* and *Hepsetus odoe* are two fishes well known in Cameroon. The chemical composition and the antibacterial activity of these fishes derived oils are unknown. The study was designed to valorise *C. nigrodigitatus* and *H.s odoe* oils activity against food poisoning bacteria.

**Methods:**

Oils were extracted by pressing and maceration methods. Their quality was assessed by analysing quality indexes including peroxides, acid, iodine, anisidine and thiobarbituric acid values. Chemical analysis was established by gas chromatography coupled to flame ionization detector. Antibacterial activity was evaluated by broth microdilution method.

**Results:**

*C. nigrodigitatus* oil obtained by maceration exhibited highest acid (7.33 ± 0.00 mg KOH/g), anisidine (34.5 ± 1.84) and thiobarbituric acid (7.50 ± 0.30 μmol MDA/Kg) values compared to that obtained by pressing method (9.13 ± 0.64 and 6.72 ± 0.34 μmol MDA/Kg) respectively. *H. odoe* oil obtained by pressing method showed highest peroxide value (6.22 ± 1.31 meq O_2_/kg). Oil chemical analysis revealed long chain polyunsaturated fatty acids of the ω-3 family: linolenic acid (C18:3); eicosapentaenoic acid (C20:5) and docosahexaenoic acid (C22:6) and ω-6 family; arachidonic acid (C20:4). In addition, *C. nigrodigitatus* oil obtained by pressing and maceration methods showed Minimum Inhibitory Concentrations (MIC) values ranging from 32 to 64 mg/ml. *H. odoe* oil obtained by pressing method revealed MIC values ranging between 8 and 64 mg/ml.

**Conclusions:**

*C. nigrodigitatus* and *H. odoe* oils have activity against food poisoning bacteria, due to their chemical composition.

## Background

Food is a source of hazards which can be either biological or chemical. Biological hazards are most frequent due to the regular ingestion of microorganisms and toxins, causing food intoxication in one out of three persons nowadays [1]. Food intoxications have a large social impact and have caused many diseases including typhoid fever, paratyphoid fever, brucellosis, botulism, cholera [2]. To limit the incidence of these diseases, antibacterial extracts including plants have been used [3]. These substances sometimes produce harmful effects. Fatty acids are natural constituents of foods with no side effect on human health. They are the main constituents of edible oils and confer to their stability countless nutritional and biological properties. Fish oils are good sources of polyunsaturated fatty acids [4]. The ω-3 and ω-6 fatty acids of fish oils are known for their therapeutic properties [5, 6]. Previous studies highlighted the antibacterial activity of polyunsaturated fatty acids from several fish oil species [5, 7]. They could be an alternative of dietary supplement against bacteria responsible for food poisoning diseases.

Cameroon is located in one of the largest river basins in the world. Its aquatic fauna is abundant and diversified. However, there are few data on fish oil composition [8] and their antimicrobial activities had almost not yet been initiated. *Chrysichthys nigrodigitatus* and *Hepsetus odoe* are widespread in the tropical regions. *C. nigrodigitatus* belongs to Claroteidae family. It is highly commercial, marketed fresh, smoked or dried, and apart from being a cheap source of highly nutritive protein, it also contains other essential nutrients required by the body [9]. *H. odoe* is usually found near the banks of rivers in heavy vegetation, but can also be found in swampy environments, lagoons and backwaters. It bears a striking resemblance to the European pike. It is however, the only member of its genus [10].

The present study was designed to promote fish oils as a complement against bacteria responsible for food poisoning diseases.

## Methods

### Fish samples

Samples of *C. nigrodigitatus* and *H. odoe* were collected in Yabassi, Cameroon. They were stored in ice and transported to the laboratory. Identification was made by ichtyologists of the Laboratory of Fisheries Resources at the Institute of Fisheries and Aquatic Sciences. The average lengths of these two fishes were 41.96 ± 9.78 cm and 31.00 ± 0.50 cm respectively for *C. nigrodigitatus* and *H. odoe*. Their average weights were 446.06 ± 67.67 g (*C. nigrodigitatus*) and 53.57 ± 3.10 g (*H. odoe*).

### Microorganisms

Microorganisms used included Gram+ and Gram- bacteria. Gram+ bacteria involved one strain of *Staphylococcus aureus* (ATCC 1026), four clinical isolates of *Staphylococcus aureus* and one clinical isolate of *Enterococcus feacalis.* Gram- bacteria included *Klebsiella pneumoniae, Salmonella paratyphi* A and *Salmonella paratyphi* S isolates.

### Oil extractions

Two techniques were used for oil extraction. The cooking-pressing method used for both fish species and maceration used for *C. nigrodigitatus*.

#### Cooking-pressing method

Twelve kilograms of each fish were washed and the undesirable parts including heads, intestines and gills were discarded. Fleshes were cooked between 80 and 85 °C in a household autoclave containing water on Kinderbo gas type plate for 20 min. The obtained fish was pressed to separate the liquid from the solid phase. The resulting pressed juice was decanted for 45 min and centrifuged 15 min at 5400 rpm/min [11]. The obtained oils were dried, weighed and stored at 4 °C until use.

#### Maceration

*C. nigrodigitatus* fleshes were dried 24 h in an oven at 45 °C. Samples were further ground to yield a fine powder. 100 g powder was mixed with 500 ml hexane, macerated and stirred three times within 24 h. Upon maceration, the homogenate was filtered and concentrated under vacuum using rotary vapour (40 °C). The resulting extract was placed in an oven at 35 °C for 2 days to remove residual solvent, weighed and stored at 4 °C until used [12].

### Determination of oil quality indexes

The peroxide value was evaluated using standard spectrophotometric method [13]. Determination of thiobarbituric acid value was done following the method recommended by American Oil Chemists’ Society [14]. The iodine value was assessed as recommended by AFNOR [15]. The acid value was determined according to standard NFT60–204 of the French Association for Standardization [15]. The anisidine value was evaluated according to AOCS Official Method Cd 18–90 “p-anisidine value” [14].

### Fatty acid profile

The fatty acid profile of oil samples were determined by gas chromatography coupled to a flame ionization detector (GC-FID). Briefly, the fatty acid methyl esters were prepared by transesterification, using 2% sulfuric acid in methanol [16]. Analysis were performed on a gas chromatograph (Agilent Technologies, Palo Alto, CA, USA), serial number 7890A, coupled to a flame ionization detector, using a capillary column DB-225 (30 m × 0.25 mm, film thickness 0.25 μm). The initial column temperature was 160 °C for 2 min, then increased to 220 °C (5 °C/min) and maintained for 10 min. Nitrogen was used as mobile phase with flow rate 1.5 ml/min. The temperature of the injector and detector were maintained at 230 °C and 250 °C respectively for 20 min. The identification of fatty acid was based on the comparison of retention time with that of standard reference fatty acid methyl esters performed under same conditions.

### Antibacterial activity

The oil’s antibacterial activity was evaluated by broth microdilution method in a 96-wells microtitre plates [17]. Bacterial suspensions of about 1.5 × 10^8^ CFU/ml (Mc Farland turbidity standard no. 0.5) was prepared from an overnight culture. The inoculum was obtained following 100 times dilution (1.5 × 10^6^ CFU/ml). The oil stock solution was prepared in 5% tween 20. A series of two fold dilutions were performed to obtain final concentration range of 0.5 to 64 mg/ml in a total volume of 100 μl/well. Each well was further diluted with 100 ml inoculum. Plates were further incubated at 35 °C for 18 h. Growth was monitored using *P-* iodotetrazolium chloride (INT; 0.2 mg/ml)) [18]. Viable bacteria change the yellow dye of iodonitrotetrazolium to a pink colour. The lowest concentration of oil, at which no visible colour change was noted was considered as MIC. Ciprofloxacin was used as positive control at concentrations ranging from 0.0625 to128 μg/ml.

### Statistical analysis

The values of the oil’s quality index were expressed as mean ± standard deviation. For a given index value, the One-Way ANOVA was used to detect differences between oil quality indexes values. When significant, the Waller Duncan test (*p* < 0.05) was used for comparison.

## Results

### Fish oil extraction yields

*C. nigrodigitatus* oil was obtained with average yields of 6.52% and 5.8% respectively by the cooking-pressing and maceration methods. *H. odoe* oil was obtained with a yield of 4.31% by cooking pressing- method.

### Fish oil chemical characteristics

#### Quality index

The quality index of oils extracted from *C. nigrodigitatus* and *H. odoe* are summarized in Table 1. The acid value of the oil extracted from *H. odoe* by cooking-pressing method was higher than *C. nigrodigitatus* oil obtained by the same method. Oils extracted from *C. nigrodigitatus* and *H. odoe* by cooking-pressing method had higher iodine values compared to *C. nigrodigitatus* oil extracted by maceration. *H. odoe*’s oil, obtained by cooking-pressing method presented the highest peroxide value. *C. nigrodigitatus* oil, extracted by maceration increases by approximately four times the anisidine value compared to that obtained by cooking-pressing method. The anisidine value of the oil extracted from *H. odoe* by cooking-pressing method was lower than that of *C. nigrodigitatus* obtained by the same method.

**Table 1:** Oil indexes of *C. nigrodigitatus and H. odoe* obtained by cooking-pressing method and maceration in hexane

Samples	Peroxyde value (meq d’O_2_/Kg)	Anisidine value	Thiobarbituric value (μmol MDA/Kg)	Iodine value (gI_2_ /100g)	Acid value (mg KOH/g)
*H. odoe* by cooking-pressing method	6.22 ± 1.31^a^	5.05 ± 0.15^c^	6.59 ± 0.07^b^	94.85 ±0,31^b^	0.98 ± 0.14^b^
*C. nigrodigitatus* by cooking-pressing method	4.49 ± 1.65^b^	9.13 ± 0.64^b^	6.72± 0,34^b^	96.28 ±0,15^a^	0.70 ± 0.14^c^
*C. nigrodigitatus* by maceration method	4.49± 0,40^b^	35.43 ± 1.84^a^	7.50 ± 0.30^a^	82.64 ±1,11^c^	7.33 ± 0.00^a^

Values are shown as mean ± standard deviation of triplicates; *n*=3. For a given oil index, values followed by different letters as superscript are significantly different, Waller Ducan test (p<0.05)

#### Fish oil chemical composition

The fatty acid profile of *C. nigrodigitatus* and *H. odoe* oils are presented in Table 2. Twenty fatty acids (C14 - C22) were identified in *C. nigrodigitatus* and *H. odoe* oils extracted by cooking-pressing method and seventeen fatty acids in *C. nigrodigitatus* oil extracted by maceration. These include saturated, monounsaturated and polyunsaturated fatty acids.

**Table 2:** Fatty acids profiles of *C. nigrodigitatus and H. odoe* oils obtained by cooking-pressing method and maceration in hexane

Acides gras	Common name of fatty acid	*C. nigrodigitatus oil* by cooking-pressing method	*C. nigrodigitatus oil* by maceration	*H. odoe oil* by cooking pressing method
C14:0	Myristic acid	4.58	2.86	1.25
C15:0	Pentadecanoic acid	1.05	0.89	0.74
C16:0	Palmitic acid	27.55	34.07	24.25
C17:0	Heptadecanoic acid	1.58	1.34	1.70
C18:0	Stearic acid	13.06	12.01	8.32
C20:0	Arachidic acid	1.73	0.34	0.76
C22:0	Behenic acid	0.38	0.21	0.42
C24:0	Lignoceric acid	0.16	/	0.15
ΣSFA		50.09	51.72	37.59
C16 :1	Palmitoleic acid	4.05	4.28	11.67
C17 :1	Heptadecanoic acid	1.07	0.76	0.69
C18 :1	Oleic acid	23.98	26.30	27.28
C20 :1	cis-11 Eicosenoic acid	1.13	2.57	0.24
C22 :1	Erucic acid	0.36	/	0.08
ΣMUFA		30.59	33.91	39.96
C18 :2	Linolelaidic acid	3.28	3.53	7.19
C18 :3	linolenic acid	1.65	1.37	3.93
C20 :2	Docosadienoic acid	0.40	0.78	1.06
C20 :3	Eicosatrienoic acid	0.57	3.01	3.84
C20 :4	Arachidonic acid	2.04	/	0.32
C20 :5	Eicosapentaenoic acid	4.10	1.29	1.45
C22 :6	Docosahexaenoic acid	6.36	2.96	2.45
ΣPUFA		18.4	12.94	20.24
ΣUFA		48.99	46.85	60.2
Σn-3		12.11	5.62	7.83
Σn-6		5.32	3.53	7.51
Σn-3 /Σ n-6		2.27	1.59	1.04

Values are expressed as percentage of fatty acid. *PUFA* polyunsaturated fatty acid, *UFA* unsaturated fatty acid, *MUFA* monounsaturated fatty acid, *SFA* saturated fatty acid

Palmitic acid was the most abundant saturated fatty acid in these three oils. *C. nigrodigitatus* oil extracted by maceration has the highest palmitic acid content (34.07%) compared to that obtained from *C. nigrodigitatus* by cooking-pressing method (27.55%) and *H. odoe* oil (24.25%).

Among the monounsaturated fatty acids, oleic acid was the most represented in the three samples. *H. odoe* oil had a higher level of oleic acid (27.28%). Cetoleic acid (C22:1) was not found in *C. nigrodigitatus* oil extracted by maceration method.

The three oils contain long chain polyunsaturated fatty acids of the ω-3 family (linolenic acid (C18:3); eicosapentaenoic acid (C20:5) and docosahexaenoic acid (C22:6)) and the ω-6 family (arachidonic acid (C20:4), linoleic acid (C18:2). Eicosapentaenoic and docosahexaenoic acids were most represented. The eicosapentaenoic and docosahexaenoic acids content in *C. nigrodigitatus* oil obtained by cooking-pressing method decreased by more than half to that obtained from the same fish by maceration. Oils extracted from *C. nigrodigitatus* had higher proportion of these two fatty acids compared to that derived from *H. odoe*. Lignoceric acid was absent in *C. nigrodigitatus* oil extracted by maceration. Arachidonic acid (C20:4) was absent in *C. nigrodigitatus* oil extracted by maceration. In general, oil extracted from *C. nigrodigitatus* by cooking-pressing method contained high levels of ω-3 fatty acids compared to that obtained from *C. nigrodigitatus* by maceration. The oil extracted from *H. odoe* contained high of ω-3 fatty acids. Similar observations were made with ω-6 fatty acids. The sum of ω-3 fatty acids contained in these three oils was greater than those of ω-6 fatty acids.

#### Fish oil antibacterial activity

Oils extracted from *C. nigrodigitatus* and *H. odoe* showed antibacterial activities on tested bacteria (Table 3). *C. nigrodigitatus* oils obtained by both cooking-pressing and maceration methods showed MICs values ranging between 32 and 64 mg/ml while *H. odoe* oil obtained by cooking- pressing method showed MICs values between 8 and 64 mg/ml. *C. nigrodigitatus* oil extracted by cooking-pressing method was active on six of the eight tested bacteria, whereas that obtained by maceration was active on four of the eight tested bacteria. *H. odoe* oil extracted by cooking-pressing method was active on seven of the eight tested bacteria. The most important activity was obtained with this oil on two of the three strains of *S. aureus* with MIC value of 8 mg/ml. Considering the whole antibacterial activity, it appears that *H. odoe* oil obtained by cooking-pressing method was the most active on the tested bacteria.

**Table 3:** Minimum Inhibitory Concentrations (mg/ml) of C. nigrodigitatus and H. odoe oils as a function of bacteria and extraction methods

	*C. nigrodigitatus oil* (cooking-ressing method)	*C. nigrodigitatus oil* (maceration)	*H. odoe oil* (cooking pressing method)	Ciprofloxacin (μg/ml)
Gram+ bacteria				
*S.aureus* ATCC1026	64	ud	64	0.125
*S. aureus* 22JN	ud	ud	8	0.125
*S. aureus* 55M	ud	ud	8	0.125
*S. aureus* 18JL	64	64	64	0.125
*E. feacalis*	64	64	ud	0.125
Gram- bacteria				
*S. parathyphi S*	32	32	64	0.125
*S. parathyphi A*	64	ud	64	0.125
*K. pneumonia*	64	64	64	0.125

*Ud* undetermined

## Discussion

The extraction yields of *C. nigrodigitatus* oils were 6.52% and 5.8% respectively, for the cooking-pressing methods and maceration. The extraction yield of *H. odoe* was 4.31% by cooking-pressing method. According to Linder et al. [19], fishes with oil content less than 5% are lean fishes, those containing 5 to 10% are semi fatty fishes and beyond 10% are considered fat. Therefore, *C. nigrodigitatus* with a yield of 5.8% - 6.52% is a semi fatty fish unlike *H. odoe* which is a lean fish (yield of 4.31%).

The acid value is a measure of the amount of free acids present in a given amount of fat. *C. nigrodigitatus* oil extracted by maceration method had the highest amount of free acids compared to that of the cooking-pressing method. This increase might be related to the extraction process. However, the acid values of *C. nigrodigitatus* and *H. odoe* oils extracted by cooking-pressing method remain in the standard (less than 3 mg KOH/g of oil) [18, 20]. This was not the case with *C. nigrodigitatus* oil extracted by maceration. The oil’s iodine value reflects its susceptibility to oxidation. A decrease is generally attributed to the destruction of double bonds, polymerization or split [21].

*C. nigrodigitatus* and *H. odoe* oils, extracted by cooking-pressing method had higher iodine values compared to *C. nigrodigitatus* oil extracted by maceration. This could be due to the fact that unsatured bonds present in *C. nigrodigitatus* and *H. odoe* oils obtained by cooking-pressing method were not affected during extraction. Therefore, the cooking-pressing method could be the best method of oil extraction of these two fishes.

Peroxide value measure the amount of hydroperoxide; the primary product of oxidized fats [22]. *H. odoe* oil extracted by cooking-pressing method showed the highest peroxide value while that extracted from *C. nigrodigitatus* showed the lowest. The high content of hydroperoxides in *H. odoe* sample compared to those extracted from *C. nigrodigitatus* revealed its deterioration caused by the alteration of unsaturated fatty acids into hydroperoxides [23].

The anisidine value reflects the secondary oxidation products, specifically 2-alkenals and 2.4-dienals unlike thiobarbituric acid value which allows highlighting the malondialdehydes which characterise the last steps of fatty acid oxidation. *C. nigrodigitatus* oil extracted by maceration presented an oxidation rate which is approximately four times higher than that of oils extracted by the cooking pressing method. Moreover, the oil extracted from *C. nigrodigitatus* by maceration exhibited the highest thiobarbituric acid value compared to the oils extracted by cooking-pressing method, reflecting last step of this oil’s oxidation. However, thiobarbituric acid values obtained with oils extracted from the two fishes regardless of the method fell within the standard range since values were below 10 μmol MDA/Kg fish [20].

From the various oil indexes, it appears that *C. nigrodigitatus* oil extracted by maceration was the most affected. Oils from *C. nigrodigitatus* and *H. odoe* obtained by cooking-pressing method showed quality indexes within the standards, meaning that they were of good quality. Nevertheless, alternative extraction methods need to be carried out in order to preserve these oils.

Oil from *C. nigrodigitatus* extracted by cooking-pressing method exhibited the lowest rate of monounsaturated fatty acids, this reflects its low rate of oleic acid (C18:1). Oil from *C. nigrodigitatus* obtained by cooking-pressing method revealed high proportions of polyunsaturated fatty acids compared to maceration. Instead, low rate of polyunsaturated fatty acid in *C. nigrodigitatus* oil obtained by maceration was observed and could be due to the absence of lignoceric, cetoleic arachidonic acid and low rates of eicosapentaenoic and docosahexaenoic acids. These fatty acids could have been oxidized and converted into hydroperoxides and malondialdehydes. This is in line with the previous result showing that the oil from *C. nigrodigitatus* obtained by maceration was more affected during the extraction process.

Oils extracted from *C. nigrodigitatus* and *H. odoe* revealed high rates of ω-3, ω-6 and monounsaturated fatty acids. The high ω-3/ω-6 constitutes their peculiarity unlike those from *Sardinella longiceps* and *Sardinella fimbriata* which are two fatty fishes from India [5].

The major compounds identified in *C. nigrodigitatus* and *H. odoe* oils were saturated fatty acids with palmitic acid being the most represented. They were followed by monounsaturated fatty acids predominantly oleic acid and followed by polyunsaturated fatty acids mostly linolelaidic acid. This repartition is quite different to that reported with the chemical composition of *Sardinella longiceps* and *Sardinella fimbriata.* Indeed, results revealed with both fishes oil composition showed that the major compounds identified were unsaturated fatty acids with a predominance of C20:5 (EPA) and C 22:6 (DHA) [5]. They are also different from *Eusphyra blochii* and *Carcharhinus bleekeri* marine fish oil where dienoic and trienoic fatty acids were found in trace amount. The findings are nevertheless similar to those reported with these marine fishes, where palmitic acid was one of the major saturated fatty acid found, oleic and palmitoleic fatty acids were the major constituents of monounsaturated fatty acids [24].

Cod liver oil is the most marketed fish derived oil for its nutritional value. EPA and DHA, its main constituent are beneficial to health. According to previous studies, they among others, ameliorate visual and cerebral functions. They are also used for regulation of some metabolic and physiological dysfunctions and have positive effects on skin, hair, nails and teeth. The oils studied also contain EPA and DHA fatty acids in significant proportions, therefore could have similar properties.

Polyunsaturated (PUFA) and monounsaturated (MUFA) fatty acids have been known to provide varied health benefits such as minimizing inflammation and/or acting as antioxidants. Recent studies highlighted n-3 PUFA and their ester derivatives antibacterial activity against various oral pathogens, including *S. mutans, C. albicans, A. actinomycetemcomitans, F. nucleatum*, and *P. gingivalis*. The major n-6 PUFAs, (linoleic acid, γ-linolenic acid, arachidonic acid), the n-7 MUFA, palmitoleic acid, the n-9 MUFA and oleic acid (OA) were found to be responsible for this activity [25].

Oils from *C. nigrodigitatus* and *H. odoe* revealed MIC values ranging between 8 and 64 mg/ml, reflecting their activities against bacteria responsible for food poisoning.

Oil composition of this two fish species were found to contain significant amounts of PUFA including linolenic acid, arachidonic acid, palmitoleic acid and oleic acid which could explain their antimicrobial activity. Similar results were earlier reported [25–28].

Oil from *H. odoe* obtained by cooking-pressing method showed activity on seven of the eight tested bacteria compared to those from *C. nigrodigitatus* obtained by cooking -pressing and maceration methods which revealed activity on six and four tested bacteria respectively. On the other hand, the best activity of *C. nigrodigitatus* oil obtained by cooking-pressing method compared to that obtained by maceration was noticed. Differences in antibacterial activity of *C. nigrodigitatus* oils could be attributed to oxidation of PUFA responsible for the activity through the maceration method. Moreover, the proportion of the PUFA responsible for the activity in the mixture may also be associated with the activity, explaining the differences between *C. nigrodigitatus* and *H. odoe* oils obtained with same procedure.

Bacteria including *S. parathyphi* A, *K. pneumoniae* and *S. aureus* are responsible for food intoxications. In addition to suffering and death, these bacteria cause considerable economic losses [29]. *S. aureus* and *S. parathyphi* A are two microorganisms responsible for food intoxication and typhoid fever respectively [30]. They were sensitive to *C. nigrodigitatus* and *H. odoe* oils, thereby indicating that these oils are potent antibacterial agents which could serve either as dietary supplements to fight against food infections caused by these bacteria or cooked in these forms to combine nutritive and curative properties of the fishes.

## Conclusion

Oils from *C. nigrodigitatus* and *H. odoe* showed significant antibacterial properties which can be justified by the presence of different classes of fatty acids including PUFA including, linolenic acid, arachidonic acid, palmitoleic acid and oleic acid. These results constitute a baseline for the exploitation of these oils as dietary supplements against bacterial infections.

## Abbreviations

ANOVA: Analysis of variance
ATCC: American type culture collection
INT: *P-* iodotetrazolium chloride
MIC: Minimum Inhibitory Concentrations
